# Relationship between triglyceride glucose-waist circumference and depressive symptoms in Chinese older adults: The China health and retirement longitudinal study

**DOI:** 10.1016/j.bbih.2026.101215

**Published:** 2026-03-17

**Authors:** Yanyan Sun, Zhifang Wang, Tingxin He, Baile Ning

**Affiliations:** a The Second Affiliated Hospital of Guangzhou University of Chinese Medicine, Guangzhou, China; b Guangzhou University of Chinese Medicine, Guangzhou, China

**Keywords:** Depressive symptoms, Triglyceride glucose, Waist circumference, The China health and retirement longitudinal study, Chinese older adults

## Abstract

**Background:**

The aim of this study was to investigate the horizontal association between triglyceride glucose waist circumference (TyG-WC) and depressive symptoms, and the underlying mechanisms.

**Methods:**

A sample of 17,708 adults aged ≥45 years from the China Health and Retirement Longitudinal Survey (CHARLS) was analyzed. Logistic regression was used to assess the impact of adverse experiences. Subgroup analysis and quartile analysis were performed simultaneously. Boruta analyzed the correlations between age and alcohol consumption and TyG-WC and depression.

**Results:**

Depressed group and non-depressed group showed considerable differences in age, education, marriage, sleep, platelet, hemoglobin, creatinine, uric acid, hypertension, heart disease, memory disorder, BMI, smoking, alcohol consumption, and stroke (*P* < 0.05). The results showed that the higher the TyG-WC level, the lower the risk of Depression. TyG-WC showed significant interactions withsex (*P*_interaction_ = 0.023) and alcohol consumption (*P*_interaction_ = 0.032), but no significant interactions with other confounding variables. Finally, analysis suggested that age and alcohol consumption were important variables associated with TyG-WC and depression(*P* < 0.05).

**Conclusions:**

Our research found that a nonlinear correlation was discovered in China between TyG-WC and depression. Factors such as age, education, marital status, sleep quality, platelet count, hemoglobin levels, creatinine levels, uric acid levels, hypertension, heart disease, memory issues, BMI, smoking habits, alcohol consumption, and history of stroke were linked to depression risk. Among these factors, alcohol consumption emerged as a significant independent variable. Detecting and addressing these risk factors promptly could potentially lower depression rates and offer significant clinical advantages to individuals.

## 1 Introduction

Depression is a major cause of the global disease burden resulting from mental disorders and has become a significant global public health issue (Mo et al., 2023). Therefore, it is important to identify specific risk factors related to depression and strive to reduce the incidence of depression.

A study reported that individuals with higher insulin resistance have more symptoms related to depression such as irritability, anhedonia, fatigue and excessive sleep, indicating that insulin resistance is associated with the occurrence of depressive symptoms (Brouwer et al., 2021). The triglyceride-glucose (TyG) index is a biochemical indicator calculated based on triglycerides and fasting blood glucose. It is regarded as one of the alternative indicators of insulin resistance and is widely used in clinical practice (Vasques et al., 2011; Yu et al., 2022). A study in the United States found that American adults with a higher TyG index have a higher risk of developing depressive symptoms, suggesting that the TyG index may serve as an indicator for predicting depressive symptoms (Shi et al., 2021). A previous cross-sectional study reported that the TyG-related indicator triglyceride-glucose waist circumference (TyG-WC) was positively correlated with depressive symptoms in premenopausal and postmenopausal women, suggesting that TyG-WC may play an important role in the occurrence of depressive symptoms in premenopausal and postmenopausal women(Liu et al., 2024). However, research on the association between TyG-WC and depression is still insufficient. Therefore, this study aims to explore the association between TyG-WC and depression and its predictive value, providing a theoretical basis for early prevention.

## 2 Methods

### 2.1 Individuals

**Fig. 1:**
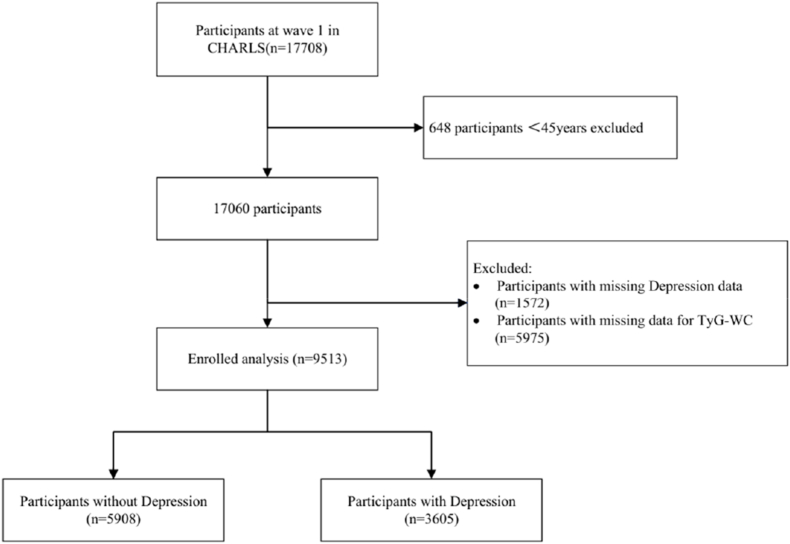
Flow chart of subject inclusion.

CHARLS is a representative follow-up survey involving people aged 45 and above in the Chinese mainland, aiming to build a high-quality public micro-database. The collected information covers multiple dimensions, such as socio-economic and health conditions to meet the needs of aging science research(Zhao et al., 2014). The data of this study are from Charls2011-2012 Wave 1, which initially included 17,708 participants. The exclusion criteria were as follows: those aged under 45 years old (n = 648), those with missing depression data (n = 1572), and those with missing TyG-WC data (n = 5975). The analysis of this study was based on the grouping of participants according to their biological sex. ,The CHARLS project website provides access to downloadable data and information.(http://charls.pku.edu.cn/).(Fig. 1)

### 2.2 Assessment of depression

Depression was defined using the 10-item short form of the Center for Epidemiologic Studies Depression Scale (CES-D-10), which has high validity and excellent psychometric properties among Chinese older individuals^]^.According to the four-classification method, the depressive state is divided into four grades: no depression (≤9), mild depression (10-14), moderate depression (15-19), and severe depression (≥20) (Irwin et al., 1999; Andresen et al., 1994). Participants were divided into the "depressive symptom group" (depressed group) and the "non-depressed group" (non-depressed group) based on the established cutoff value of CES-D-10 (≥10 points).

### 2.3 Calculation of TyG-WC

The TyG is determined through combining triglyceride (TG) and fasting glucose (FPG) data: the TyG index = ln[FPG (mg/dL) × TG] (mg/dL)/2], and the TyG-WC = TyG × WC (Dang et al., 2024; Yang et al., 2023; Wang et al., 2023)

### 2.4 Covariates

Potential confounders were selected based on prior literature and included a range of demographic, lifestyle, clinical, and health-related variables. Demographic factors comprised age, sex, marital status (categorized as married or other), and education level (classified as below primary, primary, secondary, and high school and above). Lifestyle factors included nighttime sleep duration (in hours), smoking status (yes or no), and drinking status (yes or no). Clinical indicators encompassed white blood cell count, platelet count, hemoglobin, blood urea nitrogen, creatinine, uric acid, and total cholesterol. Additionally, comorbidities were assessed based on self-reported physician diagnoses, including hypertension, heart disease, Self-reported Psychiatric disorders, and memory disorders.The sampling design, field investigation procedures, biological sample handling, and laboratory testing protocols used in this study followed the established standard operating procedures of the China Health and Retirement Longitudinal Study (CHARLS) project. Detailed documentation of these protocols is available in the official CHARLS technical reports and publicly accessible methodological publications (Mo et al., 2023; Brouwer et al., 2021).The self-reported psychiatric disorders recorded in this study specifically included depression, anxiety disorder, bipolar disorder, schizophrenia, and post-traumatic stress disorder. While such self-reported diagnoses may be subject to recall bias.they are commonly used in large-scale epidemiological surveys and have been shown to provide valid estimates of disease burden when clinical interviews are not feasible(Kelly et al., 2022). All procedures in the present analysis were implemented in strict accordance with these guidelines to ensure consistency and reproducibility(Zhao et al., 2014).

### 2.5 Statistical analysis

All statistical analyses were performed using R software. Participant characteristics were summarized using descriptive statistics, with continuous variables presented as mean (standard deviation) and categorical variables as number (percentage). Between-group comparisons were conducted using independent-samples t tests for continuous variables and χ^2^ tests for categorical variables. To evaluate the association between TyG-WC and depressive symptoms (CES-D-10 ≥ 10), multivariable logistic regression models were fitted and results were reported as odds ratios (ORs) with 95% confidence intervals (CIs). TyG-WC was categorized into quartiles (Q1–Q4), with Q1 as the reference group; Model 1 was unadjusted, Model 2 adjusted for core sociodemographic variables (age, sex, marital status, and education level), and Model 3 further adjusted for lifestyle and clinical covariates, including nighttime sleep duration, smoking status, alcohol consumption, hematological parameters (white blood cell count, platelet count, hemoglobin), renal and metabolic biomarkers (blood urea nitrogen, creatinine, uric acid, total cholesterol), and self-reported physician diagnoses/comorbidities (hypertension, heart disease, self-reported psychiatric disorders, and memory disorders). A linear trend across TyG-WC quartiles was assessed by modeling quartiles as an ordinal term. Restricted cubic spline regression was additionally applied to TyG-WC as a continuous exposure to explore potential non-linear relationships with depressive symptoms (set [number of knots and percentiles] as implemented). Prespecified subgroup analyses were conducted by age, sex, BMI, smoking, drinking, and comorbidity status, and interaction was tested by including product terms in the models. All tests were two-sided, and *P* < 0.05 was considered statistically significant.

## 3 Results

### 3.1 Characteristics of the participants

The patients were grouped according to whether they had been diagnosed with depression, with 3515depressed and 5754 non-depressed. There was no significant difference in WBC, BUN, and TC between the depression group and the non-depression group (*P* > 0.05). Still, there was a considerable difference in age, education, marriage, sleep, platelet, hemoglobin, creatinine, uric acid, hypertension, heart disease, memory disorder, BMI, smoking, alcohol consumption and stroke between the two groups (*P* < 0.05) (Table 1).

**Table 1:** Characteristics of the study population.

	[ALL]	Depressed	Non-depressed	p.overall
	*N = 9269*	*N = 3515*	*N = 5754*	
Age	59.5 (9.29)	60.4 (9.25)	58.9 (9.27)	<0.001
sex:				<0.001
Female	4932 (53.2%)	2191 (62.3%)	2741 (47.6%)	
Male	4337 (46.8%)	1324 (37.7%)	3013 (52.4%)	
Education:				<0.001
Below primary	4663 (50.3%)	2112 (60.1%)	2551 (44.3%)	
High school and above	879 (9.48%)	173 (4.92%)	706 (12.3%)	
Primary	2014 (21.7%)	721 (20.5%)	1293 (22.5%)	
Secondary	1713 (18.5%)	509 (14.5%)	1204 (20.9%)	
Marital status:				<0.001
Married	7422 (83.7%)	2654 (78.8%)	4768 (86.7%)	
Other	1447 (16.3%)	715 (21.2%)	732 (13.3%)	
Sleep duration	6.34 (1.89)	5.77 (2.10)	6.68 (1.67)	<0.001
White blood cells	6.27 (2.19)	6.26 (1.96)	6.27 (2.32)	0.837
Platelets	212 (75.7)	216 (83.7)	210 (70.3)	<0.001
Hemoglobin	14.4 (2.21)	14.2 (2.26)	14.5 (2.18)	<0.001
Urea nitrogen	15.7 (4.59)	15.7 (4.67)	15.8 (4.54)	0.657
Creatinine	0.78 (0.24)	0.76 (0.23)	0.80 (0.24)	<0.001
Uric acid	4.46 (1.25)	4.31 (1.21)	4.54 (1.27)	<0.001
Total cholesterol	194 (38.2)	195 (38.3)	193 (38.1)	0.154
Hypertension:				<0.001
no	6507 (73.4%)	2372 (70.4%)	4135 (75.2%)	
yes	2362 (26.6%)	997 (29.6%)	1365 (24.8%)	
Heart disease:				<0.001
no	7802 (88.0%)	2828 (83.9%)	4974 (90.4%)	
yes	1067 (12.0%)	541 (16.1%)	526 (9.56%)	
Self-reported Psychiatric Disorders:				<0.001
no	8763 (98.8%)	3296 (97.8%)	5467 (99.4%)	
yes	106 (1.20%)	73 (2.17%)	33 (0.60%)	
Memory disorders:				<0.001
no	8742 (98.6%)	3283 (97.4%)	5459 (99.3%)	
yes	127 (1.43%)	86 (2.55%)	41 (0.75%)	
BMI	24.1 (27.9)	23.1 (3.97)	24.6 (35.2)	0.001
Smoking:				<0.001
No	5612 (60.5%)	2287 (65.1%)	3325 (57.8%)	
Yes	3657 (39.5%)	1228 (34.9%)	2429 (42.2%)	
Drinking:				<0.001
No	6225 (67.2%)	2563 (72.9%)	3662 (63.6%)	
Yes	3044 (32.8%)	952 (27.1%)	2092 (36.4%)	
TyG_WC	735 (137)	729 (132)	739 (140)	<0.001

Depressive symptoms were defined as a CES-D-10 score ≥10. Continuous variables are presented as mean ± standard deviation, and categorical variables as number (percentage). Group comparisons were performed using independent-samples t tests for continuous variables and χ^2^ tests for categorical variables, as appropriate. All variables were assessed at baseline.

### 3.2 Non-linear association between TyG-WC and depressive symptoms

Compared to the lowest Q1 group of TyG-WC levels, the odds ratio (OR) of the highest Q4 group in the crude Model 1 was 1.32 (95% 1.18- 1.49, *P*0.001). The OR value of TyG-WC in the Q4 group was 1.34 (95% CI: 1.19-1.52, *P* < 0.001) in Model 2 and 1.41 (95% CI: 1.23-1.63, *P* < 0.001) in Model 3. The results showed that compared with the group with the lowest TyG-WC, the group with higher TyG-WC has a greater risk of depression (Table 2).

**Table 2:** Logistic regression analysis of TyG-WC and Depression in adults.

Model	Quartiles of TyG-WC				*P* for trend
	Q1	Q2	Q3	Q4	
Model 1	ref	1.13(1.32-1.56)	1.15(1.00-1.27)	1.32(1.18-1.49)	<0.001∗
Model 2	ref	1.11 (0.98-1.26)	1.15(1.10-1.30)	1.34(1.19-1.52)	<0.001∗
Model 3	ref	1.15 (1.02-1.31)	1.17(1.03-1.34)	1.41(1.23-1.63)	<0.001∗

Model 1 was unadjusted.

Model 2 adjusted for core sociodemographic variables, including age, sex, marital status, and educational level.

Model 3 was the fully adjusted model, which further included lifestyle and clinical covariates: sleep duration (nighttime), smoking status, alcohol consumption, hematological parameters (WBC, platelet count, hemoglobin), renal and metabolic biomarkers (BUN, creatinine, uric acid, total cholesterol), and self-reported history of hypertension, heart disease, psychiatric disorders, and memory disorders.

### 3.3 Subgroup analysis

Subgroups stratified by age, sex, BMI, smoking, alcohol consumption, hypertension, mental health and memory were selected to further verify the predictive ability of TyG-WC for depression. It is worth noting that TyG-WC showed significant interactions with sex (*P*_interaction_ = 0.023) and alcohol consumption (*P*_interaction_ = 0.032), but no significant interactions with other confounding variables (Table 3).

**Table 3:** Subgroup analyses of the association between TyG-WC and depressive symptoms.

Variable	Count	Percent	Point Estimate	Lower	Upper	*P* value	*P* for interaction
Overall	9269	100	0.91	0.87	0.95	<0.001	
Age							0.938
≥60	4258	45.9	0.91	0.86	0.97	0.003	
45-60	5011	54.1	0.91	0.86	0.97	0.003	
Gender							0.023
Female	4932	53.2	0.93	0.88	0.98	0.011	
Male	4337	46.8	0.85	0.79	0.9	<0.001	
Bmi							0.998
<25	6360	68.6	0.96	0.91	1.01	0.128	
≥25	2909	31.4	0.96	0.89	1.04	0.351	
Smoking							0.247
No	5612	60.5	0.92	0.87	0.97	0.001	
Yes	3657	39.5	0.88	0.82	0.94	<0.001	
Drinking							0.032
No	6225	67.2	0.93	0.88	0.98	0.004	
Yes	3044	32.8	0.85	0.78	0.91	<0.001	
Hypertension							0.932
No	6507	73.4	0.89	0.84	0.93	<0.001	
Yes	2362	26.6	0.87	0.81	0.95	0.001	
Heart disease							0.884
No	7802	88	0.89	0.85	0.93	<0.001	
Yes	1067	12	0.87	0.77	0.98	0.021	
Self-reported psychiatric disorders							0.472
No	8763	98.8	0.91	0.87	0.95	<0.001	
Yes	106	1.2	0.77	0.5	1.17	0.216	
Memory disorders							0.392
No	8742	98.6	0.9	0.86	0.94	<0.001	
Yes	127	1.4	1.07	0.74	1.57	0.706	

Subgroup analyses of the association between TyG-WC (per standard deviation increase) and depressive symptoms. Results are presented as ORs with 95% CIs. Subgroups were defined according to age, sex, BMI, smoking status, drinking, and comorbidity status. Interaction *P* values were obtained by including multiplicative interaction terms in the fully adjusted model.

### 3.4 Linear relationship between TyG-WC and depression

**Fig. 2:**
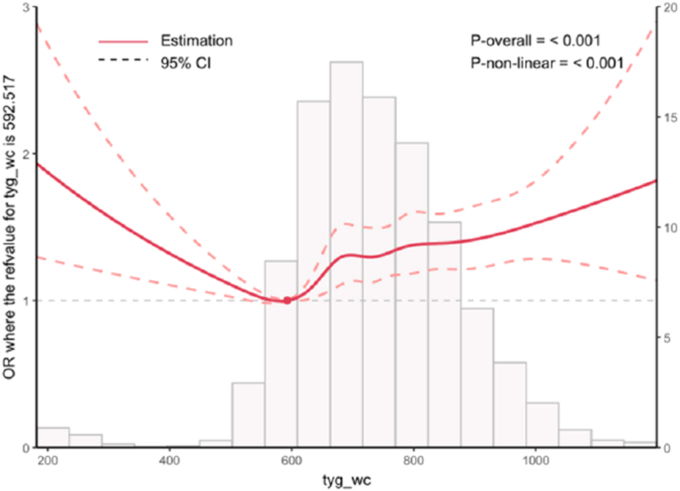
RCS analysis between TyG-WC and depression3.5 Relationship between TyG-WC and depression Note:Core sociodemographic variables were adjusted, including age, sex, marital status, educational level, sleep duration (nighttime), smoking status, alcohol consumption, hematological parameters (white blood cell count, platelet count, hemoglobin), kidney and metabolic biomarkers (urea nitrogen, creatinine, uric acid, total cholesterol). And the self-reported history of hypertension, heart disease, self-reported psychiatric disorders and memory disorders followed by the TyG-wc and depression RCS curves. Tyg-wc is triglyceride-glucose waist circumference.OR is Odds Ratio.

To further explore and visualize the relationship between TyG-WC and depression, RCS curves were plotted and adjusted for all covariates. The results showed that TyG-WC was significantly correlated with the risk of depression (*P* < 0.001), showing a nonlinear U-shaped correlation (nonlinear Pnonlinear = 0.018) as shown in the figure(Fig. 2).

**Fig. 3:**
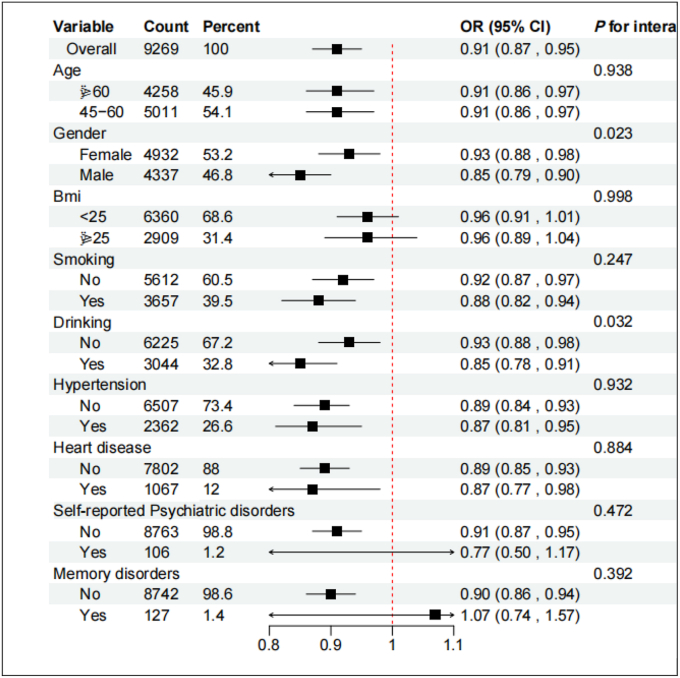
Interaction Analysis of TyG-WC and Depression Note: OR is Odds Ratio.

In order to better show the association between TyG-WC and depression, collinearity analysis was performed first, followed by variable screening using Boruta analysis. The date shows that age and alcohol consumption were important variables associated with TyG-WC and depression(*P* < 0.05)(Fig. 3).

## 4 Discussion

This study extends the existing literature on metabolic indicators and depression in several key ways. First, whereas prior research has predominantly examined linear associations of the Triglyceride-Glucose (TyG) index with depression(Liu et al., 2024). We are the first to identify a significant nonlinear relationship between the TyG-Waist Circumference (TyG-WC) index and depressive symptoms specifically among adults aged 45 years and older. Second, our subgroup analyses revealed that gender and alcohol consumption significantly modified this association, suggesting a potential synergistic interaction between these lifestyle and biological factors in influencing depression risk. Third, these findings collectively provide novel epidemiological evidence, underscoring the importance of integrating anthropometric measures (like waist circumference) and considering effect modifiers in the study of depression-related metabolic pathways.

This study aimed to investigate the association between TyG-WC and depression in a nationally representative sample from the Charles dataset. The main finding of this paper is that high levels of TyG-WC are associated with a higher risk of depression in Chinese adults. After different stratification analyses and sensitivity analyses, the results remained consistent. By further verifying the predictive ability of TyG-WC for depression, stratified analysis of subgroups based on age, sex, BMI, smoking, alcohol consumption, hypertension, mental health and memory showed that TyG-WC had significant interaction with sex and alcohol consumption, which was consistent with the conclusions of previous studies. Age and alcohol consumption were significant variables associated with TyG-WC and depression.

Thus, the mechanistic details of the TyG-WC index's association with depression may have several possible explanations. First, depressed patients have poor IR. With increased insulin resistance, the risk of depression increased by 4% in younger adults and 17% in non-diabetic patients(Lee et al., 2017). In this study, a positive correlation between the TyG-WC index and depression was observed in a large population of China. The results of this study were similar to those previously published and the association between TyG-WC and depression remained consistent even across subgroup analyses. Second, the association between high levels of TyG-WC and higher odds of depression was strongest in models adjusted for age, sex, and alcohol consumption and decreased when adjusted for baseline comorbidities(Shi et al., 2021). Third, alcohol-induced inflammation may play an important role in the association between TyG-WC index and depression. Previous studies have shown that the TyG-WC index is positively correlated with inflammatory markers of leukocytes and C-reactive protein (Huta-Osiecka et al., 2022; Wan et al., 2025; Nam et al., 2020). Previous studies have suggested that the inflammatory response to depressive mood in patients with depression is closely related, and the inflammatory response can interact with the patients' own depressive mood (Zhou et al., 2021). Previous studies have shown that the levels of inflammatory factors in patients with depression change significantly, suggesting that the pathogenesis may be related to neuroimmune responses(Cacabelos et al., 2016). Multiple studies suggest that the release of inflammatory mediators affects the synthesis and release of neurotransmitters in the brain, leading to depressive symptoms. Therefore, neuroimmunity may be involved in the pathophysiological process of depression(Hassamal, 2023). Smith et al. discovered in 1991 that macrophages are involved in the occurrence and development of depression, and pro-inflammatory factors are also involved in interfering with the normal regulatory activities of hormones in patients with depression(Smith, 1991). In conclusion, inflammation is involved in the occurrence of depression.immunometabolic depression (IMD) refers to the aggregation of inflammatory and metabolic disorders and atypical energy-related symptoms (AES) in patients with depression, and has not yet been defined as a clinical subtype of depression. This is a potential cross-diagnostic concept whose immunometabolic characteristics also exist in various psychologies (such as bipolar disorder, anxiety disorder) or physical disorders (such as obesity, diabetes). In a previous study, it was found that the higher the IMD index (based on AES severity, body mass index and C-reactive protein) score, the smaller the reduction in the severity of depressive symptoms during antidepressant treatment (Vreijling et al., 2024, 2025).Alcohol damage can lead to abnormalities in white blood cells and C-reactive protein in the body, leading to metabolic abnormalities and nerve damage, and alcohol can also cause abnormalities in TyG-WC (Wakabayashi, 2023; He and Huang, 2024; Keller et al., 2024; Kalra and Raizada, 2024).In previous studies, alcohol consumption was a risk factor for depression (Nielsen and Andersen, 2022).

Our results are important for further understanding the relationship between insulin resistance and depression, especially in the general population. TyG-WC represents IR accompanied by lipid or glucose metabolism disorder, which is consistent with the glucose fatty acid cycle hypothesis(Vasques et al., 2011). Depression patients had higher triglycerides and lower HDL cholesterol levels than healthy controls. Both WC and TyG have been previously linked to depression, and we need to further explore the common mechanisms between them to help block the effects of depression. A better understanding of physiology may open the way to specific treatments for depression. The results showed differences in depression, alcohol use, and age between healthy and ill patients. We acknowledge that it may have selection bias, but we model it by adjusting for different variables. The orientation of the model is consistent. Although there are some defects in sample selection, our conclusions are still reliable. This study provides a basis for future multicenter cohort studies of depression and TyG-WC.

Sex differences play an important role in the relationship between depression and TyG-WC. Studies have shown that the correlation between female TyG-WC and depressive symptoms is stronger (Liu et al., 2025). This sex difference may be related to the synthesis, metabolism and receptor function of monoamine neurotransmitters. The 5-hydroxytryptamin (5-HT) system in women is more prone to damage: the synthesis rate of 5-HT in their cerebral cortex is lower than that in men, and the reduction of 5-HT is more significant after tryptophan depletion(Veral et al., 1997). This further exacerbates the risk of depression. This sex difference may be attributed to hormonal influences, such as the role of estrogen in lipid metabolism and mood regulation, the higher incidence of visceral obesity in postmenopausal women, and the excessive impact of psychosocial stress on women(Gado et al., 2024). To sum up, the association between TyG-WC and depression is more obvious in women.

However, this study has some limitations. First, cross-section does not imply causality. The population included in this study is China, and it cannot be directly extrapolated to other ethnic groups, such as Caucasian or Black, so the results are mainly applicable to China. Second, the data do not completely rule out the effects of medications taken by participants for other chronic conditions on depression and lipid and glucose metabolism disorders. Finally, the main limitation of this study lies in the limited data in the CHARLS database, which makes it impossible for us to obtain detailed clinical diagnosis records and comprehensive medication history (especially for psychotropic drugs and specific metabolic drug types). Although we included multiple self-reported disease, functional status indicators, and treatment status variables and conducted a rigorous subgroup analysis to mitigate the potential confounding bias resulting from this, the possibility of residual confounding still could not be completely ruled out. The ideal future research needs to combine community epidemiological investigations with detailed medical records or biobank data to verify the findings of this study after more strictly controlling for clinical confounding factors. Therefore, it cannot be used as an exact causal relationship, but it can provide more evidence for studying risk factors for depression and help research on the disease.

## 5 Conclusions

Our research found that a nonlinear correlation was discovered in China between TyG-WC and depression. Factors such as age, education, marital status, sleep quality, platelet count, hemoglobin levels, creatinine levels, uric acid levels, hypertension, heart disease, memory issues, BMI, smoking habits, alcohol consumption, and history of stroke were linked to depression risk. Among these factors, alcohol consumption emerged as a significant independent variable. Detecting and addressing these risk factors promptly could potentially lower depression rates and offer significant clinical advantages to individuals. Consistent with previous studies(Sun et al., 2025).

## CRediT authorship contribution statement

**Yanyan Sun:** Writing – review & editing, Writing – original draft, Supervision, Conceptualization. **Zhifang Wang:** Writing – original draft, Conceptualization. **Tingxin He:** Writing – original draft, Conceptualization. **Baile Ning:** Writing – review & editing, Writing – original draft, Supervision, Project administration, Conceptualization.

## Data access

The data that support the findings of the study are available through the website of CHARLS: http://charls.pku.edu.cn/index/en.html. To access and use the day for research purpose, approval should be ob tained from the CHARLS team at Peking University.

## Role of the funding source

This work was supported by the 10.13039/501100001809National Natural Science Foundation of China (grant number: 82474620); The Guangdong Province Natural Science Foundation-Youth Promotion Project (grant number: 2024A1515030202); and The Traditional Chinese Medicine Scientific Research Project of Guangdong Province Bureau of Traditional Chinese Medicine (grant number: 20251137).

## Declaration of competing interest

The authors declare that the research was conducted in the absence of any commercial or financial relationships that could be construed as a potential conflict of interest.

## Data Availability

Data will be made available on request.

## Abbreviations

CHARLS: The China Health and Retirement Longitudinal Study
TyG-WC: triglyceride glucose waist circumference
IR: Insulin resistance
TyG: Triglyceride glucose
CES-D-10: Epidemiologic Studies Depression Scale
TG: triglyceride
FPG: fasting glucose
